# Buffalo Medical and Surgical Association

**Published:** 1880-10

**Authors:** 


### BUFFALO MEDICAL AND SURGICAL ASSOCIATION.

*Stated Meeting, September 2, 1880.*

THE PRESIDENT, DR. LUCIEN HOWE, IN THE CHAIR.

After the reading and approval of the minutes of the last meeting, Dr. Granger presented the paper for the evening on "State Regulation of the Practice of Medicine." [Extracts from this paper form the first article in the present number of this Journal.]

In the discussion which followed, Dr. Rochester said he agreed with the writer in his view of the subject and only hoped that the law in this state, which was about to go into operation, would be found practicable in its working.

Dr. Cronyn spoke with special reference to the apparent ambiguity of the last section of this law. He quoted the interpretation of the State Medical Society to the effect that it was no exemption from registration that we have practiced for ten years past. According to this, we must register whether we have practiced or not.

Dr. Bonner, present by invitation, referred to a similar system in vogue in Canada, in spite of which, however, several men with diplomas from disreputable schools had, in some way, succeeded in obtaining a license to practice.

Dr. Wyckoff expressed his gratification as to the existence of such a law, but regretted any misunderstanding should be possible as to its interpretation.

Dr. White thanked the writer of the paper for calling attention to the subject. He thought, however, that the nature of our institutions was such, as to render legislation inoperative unless public sentiment could be educated to sustain the laws. These must originate with the community as a whole. If reputable physicians assist in forming medical laws, the ignorant and the quacks at once raise a cry against "class legislation."

If we ask for enactments regulating the practice of medicine, therefore, we arouse the suspicion that we are trying to benefit ourselves, instead of the public, for which such laws are really made. The only defense for honest physicians is in the medical societies. To these we can admit only those with whom we wish to be associated. In regard to the ambiguity as to the necessity of registering, as expressed in the law recently passed, Dr. White thought the Association should be enabled to act intelligently and to this end he proposed the following resolutions:

First, "That when this Association adjourns, it do adjourn to meet a fortnight from this evening, to hear a report from a committee which shall be appointed for the purpose of obtaining a legal opinion in regard to this law, and

Second, "That Drs. Granger, Barker and Keene be constituted a committee, and empowered to obtain from Judge Clinton, or some other gentleman of the bar, a clear interpretation of this law for our guidance."

These motions being separately seconded and voted upon, were both carried.

Under the head of "Voluntary Communications" Dr. Barker gave the history of a case of strangulation of the small intestine by the mesentery of the Ileum, (which appears as a Clinical Report in this issue of the Journal,) and at the same time presented for inspection a portion of the part involved. It was highly injected, much thickened, and distended villi of unusually large size covered its entire inner surface.

Dr. Howe called Dr. Rochester to the chair, and directed the attention of the Association to a form of demonstrating ophthalmoscope, by which the size of the retinal image could be rapidly made larger or smaller according as the observer desired a view of the fundus in general, or in detail. In this, too, the source of light was so protected as to cause not the least inconvenience to the patient, even when the examination was long continued The working of the instrument was illustrated upon a case of partial atrophy of the optic nerve.

As a second communication Dr. Howe referred "to some experiments upon rabbits with a view to inoculate their conjunctivae with gonorrhœal virus. The attempts were not sufficiently satisfactory to warrant any definite conclusions, and a request was made for assistance in obtaining the virus in different stages, from patients having that disease.

Reports from the different members as to prevailing diseases were meagre, there being in general less dysentery, diarrhœa, and similar troubles, than are usually met with at this season.

After transacting some routine business the Association adjourned.

SPECIAL MEETING, SEPT. 7.

The President was in the chair and the following were present: Drs. White, Rochester, MacNeil, Cronyn, Samo, Green, Mynter, Kilbourne, Keene, Bartlett, Hauenstein, Cary, Barker, Bailey, Shaw and Granger.

The PRESIDENT, in stating the object of the meeting, said that the exact meaning of the recent enactment was not entirely clear, and referred to the manner in which a discussion at the last meeting had led to the appointment of this committee.

DR. GRANGER then presented the following report on the interpretation of the medical law of 1880, chapter 513:

The Committee respectfully report that they have procured the written opinion of Judge George W. Clinton upon the subject referred to them. The Committee find no reason to question the correctness of Judge Clinton's interpretation of section 6 of the law, which is the most important point in the law to be determined. We therefore unanimously advise all members of this Association, whether they have been in practice a longer or shorter time than ten years, to register at the County Clerk's Office before the 2d day of October next.

WM. D. GRANGER,
A. M. BARKER,
J. W. KEENE,
Committee.

With the report the following opinion, by Judge Clinton, was presented and read:

OPINION.

Chapter 275 of the act of 1844 (sections 1 and 2) repealed all laws of the State prohibiting any person from receiving compensation for medical services, and provided that persons practicing without a license should not be liable to criminal punishment for practicing except in cases of malpractice, gross ignorance, or immoral conduct. It evidently divided all practitioners into two classes—the legally authorized and unauthorized. No one could be punished for practicing medicine without a license—he was enabled to recover the worth of his services, and was liable to severe punishment for malpractice, or gross ignorance, or immoral conduct. This distinction remained untouched until the passage of chapter 513 of the act of 1880. By section 1 that act abolishes all the privileges and exemptions of the before legally unauthorized class of practitioners. It prohibits a person not authorized by the act from practicing medicine and surgery. Consequently, such person, if he practice medicine or surgery, cannot maintain an action for his services; and the third section of the act makes him criminally liable to fine or imprisonment for so practicing. These evident changes in the general laws touching the practice of medicine and surgery sufficiently explain, in my opinion, the exception embodied in the concluding sentence of section 6 of the act. It was felt to be unjust that an unlicensed person who had practiced for ten years "last past" continuously without subjecting himself to punishment for malpractice or gross ignorance or immoral conduct, and who was actually pursuing the study of medicine and surgery in a legally incorporated medical college of the State, should be at once made punishable criminally for practicing, and therefore the exemption was enacted which allows him to continue his practice, subject to the old law, until he graduates and receives a diploma or is denied graduation within two years from the passage of the act.

If we erect the clause, "Nor shall there be any person who has practiced medicine and surgery for ten years last past" into a distinct sentence, the rest of the clause becomes sheer nonsense. The sentence, as it stands, must be read as one complete sentence, and so read, it cannot apply to any regular physician and surgeon of ten years' standing. No such physician "is now pursuing the study of medicine and surgery in" any "legally incorporated college in this State," with the intent to graduate from it and receive a diploma within two years. My opinion is that every person legally qualified to practice medicine and surgery in this State, who does not register himself in compliance with the act, (with the exception of section six), is prohibited from practicing and made criminally liable by section three for practicing his profession. Our County Clerk has provided a book for registrations, consisting of blank affidavits. When a physician has filled the blank and taken the oath, there can be no doubt that he has fully complied with the law.

BUFFALO, Sept. 15, 1880. G. W. CLINTON.

DR. ROCHESTER moved that the report be accepted and the committee discharged. Carried.

DR. S. C. GREENE called attention to the fact that the law did not make it the duty of anyone to prosecute persons violating the law, and suggested the law should be amended to make it the special duty of the District Attorney to prosecute.

DR. ROCHESTER said that an inducement to prosecute was made in the law by allowing persons making the complaint one-half the fine imposed. In reply to a question Dr. Rochester said he should consider it very dishonorable for anyone to prosecute simply to make money out of it.

DR. WHITE feared the law would be inoperative in driving out quacks, and that the people must sustain the law; that it would raise the cry of persecution should the regular physicians alone attempt to enforce the law.

DR. CRONYN thought that the District Attorney was too busy to do the work of prosecuting unless complaints were made in the regular way and came to him as other criminal cases did, to try. He suggested that a person be appointed whose special duty it should be to institute proceedings against violators of the law.

DR. GRANGER said that it seems an act of courtesy, as we now had an accepted interpretation of the law, to inform other physicians, not members of this Society, of the opinion we had obtained; and, also, for the purpose that the subject of enforcing the law might be brought before the county society, offered the following resolutions:

*Resolved*, That the Secretary be requested to inform the Secretary of the Erie County Medical Society of the true meaning of the new medical law, as interpreted to us by Judge Clinton, and request him to inform all members of that Society of the necessity to comply with the requirements of the law before October 2, 1880.

*Resolved*, That the Secretary be further requested to bring this law to the notice of the Erie County Society at its next meeting, and request them to take such measures as shall seem best to them to enforce the law against all unauthorized practitioners in this county.

Dr. White said that as all must register before October 2d, it might be that all members of the Erie County Society would not be informed of the true meaning of the law in time to register, therefore he moved the following resolution:

*Resolved*, That the Committee on the New Medical Law be reappointed and be requested to procure the publication of the proceedings of this Society in relation to the interpretation of the medical law passed at the season of 1880; and that they be requested to send a copy to every member of the Erie County Medical Society, and to the Secretary of the Medical Society of the Eighth Judicial District.

Seconded by Dr. Greene. Carried.

Adjourned.

